# Enhancing health professional clinical performance through supervision strategies: from 5311 facilities across eight countries in sick child healthcare

**DOI:** 10.7189/jogh.16.04123

**Published:** 2026-04-24

**Authors:** Mengyao Li, Zhongliang Zhou, Rebecca Mitchell, Dan Wang, Brendan Boyle, Wenhua Wang

**Affiliations:** 1 School of Public Policy and Administration, Xi’an Jiaotong University, Xi’an, China; 2 Health at Work Research Centre and Macquarie Business School, Macquarie University, North Ryde, New South Wales, Australia; 3 Newcastle Business School, University of Newcastle, Newcastle, New South Wales, Australia; 4 School of Pharmaceutical Sciences, Wenzhou Medical University, Wenzhou, China; 5 Institute of Healthcare Innovation and Intelligent Governance, Wenzhou Medical University, Wenzhou, China

## Abstract

**Background:**

Supervision remains a widely implemented strategy to improve clinical quality, but its independent impact and the means to enhance its effectiveness remain uncertain. We investigated the effectiveness of supervision alone and, in combination with feedback or training, on clinical quality in child healthcare, and explored the relationship between supervision alone and performance across different organisational contexts.

**Methods:**

Data were sourced from the Service Provision Assessment survey, covering 5311 health facilities, 6722 health providers, and 20 880 sick children across eight low- and middle-income countries (LMICs). We assessed health worker performance using 20 clinical quality indicators. We examined various supervision modalities, including supervision alone, supervision with feedback, and supervision with training. We considered organisational factors, such as quality assurance activities and data collection systems and applied multilevel regression models, adjusting for relevant covariates.

**Results:**

Supervision alone was not significantly related to clinical performance (coefficient = 0.101; 95% confidence interval (CI) = –0.00, 0.20). However, supervision combined with feedback (Coefficient = 0.171; 95% CI = 0.07, 0.27) or training (coefficient = 0.514; 95% CI = 0.41, 0.62) were both significantly associated with better performance. Organisational support, particularly through quality assurance activities (coefficient = 0.290; 95% CI = 0.14, 0.44) or data collection systems (coefficient = 0.130; 95% CI = 0.02, 0.24), transformed otherwise ineffective supervision into effective ones. The positive impact was stronger when both quality assurance and data collection systems were present (coefficient = 0.319; 95% CI = 0.17, 0.47).

**Conclusions:**

Supervision alone is inadequate in relation to better clinical quality in sick child healthcare services. A combination of supervision strategies, including feedback and training, along with institutional support, is likely to be associated with better health worker performance, particularly in LMICs. These findings highlight the need for integrated approaches to supervision, emphasising the role of institutional support in driving quality improvements in child health services.

The quality of care delivered by health professionals is essential for achieving universal health coverage, yet remains suboptimal, especially in low- and middle-income countries (LMICs) [1,2]. Poor quality of care significantly contributes to preventable morbidity and mortality, resulting in an estimated five million deaths annually and projected cumulative economic losses of USD 11.2 trillion globally between 2015 and 2030 [3,4]. The burden is disproportionately high in LMICs, where healthcare systems often face resource constraints, and existing quality improvement efforts have seen limited success [5]. Among these efforts, the supervision of health professionals is one of the most commonly implemented interventions to improve clinical practices and overall quality of care.

Supervision varies widely across disciplines and contexts, complicating its definition and scope [5]. In the health sector, it refers to the organisational and management process that strengthens the health system, enhances provider practices, and improves performance [6]. Various terms, such as professional supervision, clinical supervision, managerial supervision, and support supervision, are often used interchangeably in the literature [7–10]. Historically, traditional supervision followed a top-down approach, a hierarchical model, where senior personnel monitored junior staff to ensure compliance with standards [11,12]. Over time, traditional supervision has evolved into a more collaborative model of ‘support supervision,’ emphasising joint problem-solving and motivation rather than mere oversight, and is not restricted by specific subjects or timeframes [5,11]. Qualitative research has underscored the potential of supervision to enhance staff motivation and job satisfaction [13]; however, its direct effects on clinical practice and quality of care remain less clear [5].

Empirical findings on the impact of supervision on health professionals’ performance and clinical outcomes have been mixed. Some studies reported that supervision could enhance provider capacity and improve clinical practice [14–18]. However, other research indicated that supervision, when implemented in isolation, did not affect nursing [7] or maternal healthcare [19]. Even the effect of supervision frequency was contested: while one study indicated that reducing supervision frequency did not compromise the quality of care [20], another suggested that more frequent supervision improved midwifery performance [21]. These inconsistencies might stem from the fact that supervision was often implemented alongside other interventions. Common strategies combined with supervision included training, audit with feedback, and peer review, *etc.* [16,18,22–25]. In these mixed strategies, it becomes difficult to isolate the specific effects of supervision on clinical quality and identify the key elements that make supervision effective.

Effective supervision depends not only on its characteristics and individual-focused modalities but also on external and organisational factors, which often receive insufficient attention. Research has highlighted that policy direction [26,27], organisational culture [27,28], and organisational support [28]. They are key elements in ensuring the successful implementation of supervision interventions. However, while these organisational factors are important, environmental factors identified in the remote supervision study focus on individual-level changes, like access to telephones or computers [29], rather than the facility-level improvements. At the organisational level, poor-quality supervision could stem from excessive focus on service statistics during sessions at the expense of clinical care improvements [20]. Additionally, robust quality assurance mechanisms are essential for improving the sustainability of supervision interventions [6,30]. Despite their importance, quantitative evidence on the impact of data collection systems and quality assurance activities on supervision implementation, particularly regarding the interplay of supervision modalities and organisational contexts, remains limited. Addressing these gaps is crucial for optimising supervision at both institutional and individual levels to enhance clinical quality.

In this study, we aimed to address these gaps by leveraging data from the Service Provision Assessment (SPA) survey. Initially, we assessed the relationship between supervision alone and the quality of care delivered by health professionals. Next, we analysed how supervision modalities, when combined with other targeted strategies aimed at individual health professionals, are associated with clinical quality. Finally, we examined how the impact of supervision alone on clinic quality varied across different organisational contexts. By examining these relationships, this study seeks to generate robust evidence on optimizing supervisory interventions at the institutional and individual levels to enhance clinical quality. These findings will provide valuable information for policymakers and healthcare administrators to design and implement more effective supervisory interventions, with a particular focus on improving practices in LMICs.

## METHODS

### Data sources and study sample

We utilised data from the SPA, which is a part of the Demographic and Health Survey program. The SPA survey primarily examines the capacity of health facilities, the performance of health providers, and patients’ reported experiences of service utilisation. This global survey offers nationally representative data and includes facility inventories, health worker interviews, and client exit interviews [31]. The SPA survey provides detailed insights into the clinical performance of health professionals and the strategies implemented to improve it, targeting health providers or health facilities and aligning well with the objective of our study.

For the analyses, we used data on sick-child healthcare services and health facilities. Since the SPA survey is not conducted annually in every country, we selected the most recent data from the past 10 years to reflect approximate contemporary conditions. We excluded one country that did not survey sick child healthcare services. Ultimately, data from eight countries were included in the analysis: Afghanistan (2019), the Democratic Republic of Congo (2018), Ethiopia (2022), Haiti (2018), Malawi (2014), Nepal (2021), Senegal (2019), and Tanzania (2015). The sample comprised 5311 health facilities, 6722 health providers, and 20 880 sick children.

### Clinical quality measurement

We defined clinical quality as the extent to which health providers delivered services to clients in accordance with clinical guidelines, reflecting the overall quality of care provided by health professionals. We derived 20 indicators from the international guideline and used them to measure clinical quality [32]. These indicators encompassed the entire standard visit process, including systematic assessment, diagnosis, treatment, and counselling. Each indicator was observed by an interviewer, and health professionals received one point for each completed indicator, with a maximum score of 20 points. Higher scores indicated better clinical quality. To ensure the robustness of the results, we also utilised 10 indicators from another study to measure clinical quality [33]. The scoring method remained the same as in the previous version, with a total score ranging from zero to 10, where higher scores indicated better clinical quality (Appendix S1 in the **Online Supplementary Document**).

### Supervision measurement

Supervision strategies in this study encompassed modalities for individual health professionals and supportive institutional contexts. The modalities of supervision included: supervision alone, supervision with feedback, and supervision with training. Supervision alone refers to monitoring and assessing health professionals without additional interventions. Supervision with feedback and supervision with training are categorised as optimised supervision modalities. Specifically, supervision with feedback involves providing feedback, either verbally or in writing, on health professionals’ performance. Supervision with training combines supervision with targeted in-service training designed to improve specific competencies, such as those related to child health. These two optimised modalities offer additional support and development opportunities for health professionals, moving beyond simple monitoring to enhance their skills and performance. The detailed measurements for these modalities are as follows: Supervision was measured by asking health professionals, ‘Have you received supervision in the past six months, either from a supervisor within the facility or from outside the facility?’ Feedback was assessed with the question, ‘Did your supervisor provide any feedback on your performance, either verbally or in written form?’ Training was measured by asking, ‘Have you received any in-service training on topics related to child health or childhood illnesses?’

Organisational contexts included two key variables – quality assurance and data collection systems. We assessed quality assurance by asking: ‘Does the facility routinely carry out quality assurance activities?’ The data collection system was measured with the question: ‘Does this facility have a system in place to regularly collect health services data?’ We grouped facilities based on whether they implemented quality assurance activities, data collection systems, or both.

### Covariates

Based on previous literature, we identified four levels of control variables that could potentially impact clinical quality [22,33,34]. The covariates included the sick-child, caretaker, health-provider, and health-facility levels. At the sick child level, variables included sex and age. The caretaker level comprised the caretaker’s age and their relationship to the child (mother or non-mother). The health provider level included the type of provider, years of service at the current facility, weekly working hours, years of education, and whether they perceived their work environment as supportive. The health facility level considered ownership (government or private), location (urban or rural), and facility type (hospital or clinic). All covariates were incorporated into the regression model to minimise potential bias.

### Data analysis

First, we conducted descriptive analyses to present the basic characteristics of health facilities, health providers, caretakers, and sick children from the sample of eight countries. We used frequencies and percentages to describe categorical variables, and means and standard errors for continuous variables. Second, we analysed clinical quality under different supervision modalities or organisational contexts. A figure illustrated the varying levels of clinical quality under various supervision modalities, as well as the levels of clinical quality depending on whether health facilities had implemented the context variables.

Next, we used multilevel regression models to test the relationship between supervision and clinical quality, adjusting for covariates. Initially, we tested the relationship between supervision alone and clinical quality. Next, we examined two modalities of supervision targeting individual health professionals, exploring the relationship between clinical quality and supervision with feedback, and supervision with training. Additionally, we conducted a subgroup analysis of the multilevel regression model to examine the relationship between supervision alone and clinical quality, depending on whether the health facility had a specific context (quality assurance activities, data collection systems, or both).

Finally, we replaced the clinical quality measure with an alternative measure to assess the robustness of the main results, using the same regression methods. It was important to note that all statistical analyses were unweighted, as the objective of the study was to examine relationships rather than produce nationally representative estimates that reflect the characteristics of any single country. To account for potential cross-country heterogeneity, we included country fixed effects in all regression models. This approach helped control for unobserved, time-invariant country-level factors that may influence clinical quality. For all analyses, we used Stata, version 17.0 (StataCorp, College Station, Texas, USA).

## RESULTS

Over 80% (n = 16 758, 80.98%) of the sick children were cared for by their mothers during visits to the health provider. On average, health providers had worked at their current facility for 6.17 years and worked 50.85 hours per week. They had an average of 16.40 years of education, and 35% reported a supportive environment in their health facility. Regarding health facilities, the majority were located in rural areas (n = 2997, 56.43%), were government-managed (n = 3467, 65.28%), and were classified as clinics (n = 3725, 70.14%) (**Table 1**).

**Table 1:** Characteristics of health facilities, health providers, caretakers, and sick children in the sample size from eight countries

	n (%)
**Facility (n = 5311)**	5311
Location	
*Urban*	2314 (43.57)
*Rural*	2997 (56.43)
Manage authority	
*Government*	3467 (65.28)
*Private*	1844 (34.72)
Type	
*Hospital*	1586 (29.86)
*Clinic*	3725 (70.14)
**Provider (n = 6722)**	
Type	
*Physician*	3399 (51.01)
*Nurse*	3265 (48.99)
Years of working, x̄ (SD)	6.17 (6.25)
Working hours per week, x̄ (SD)	50.85 (18.87)
Years of education, x̄ (SD)	16.40 (3.35)
Support environment, x̄ (SD)*	0.35 (0.48)
**Caretaker (n = 20 880)**	
Relationship to child	16 758 (80.98)
Mother	3936 (19.02)
Non-mother	28.71 (7.88)
**Child (n = 20 880)**	
Age in months, x̄ (SD)	20.47 (15.59)
Sex	
*Male*	11 095 (53.14)
*Female*	9782 (46.86)

SD – standard deviation, x̄ – mean

*Supportive environment is a binary indicator that contains at least two of the following three elements: knowledge of advancement opportunities, well-defined job descriptions, and extra performance incentives.

**Figure 1:**
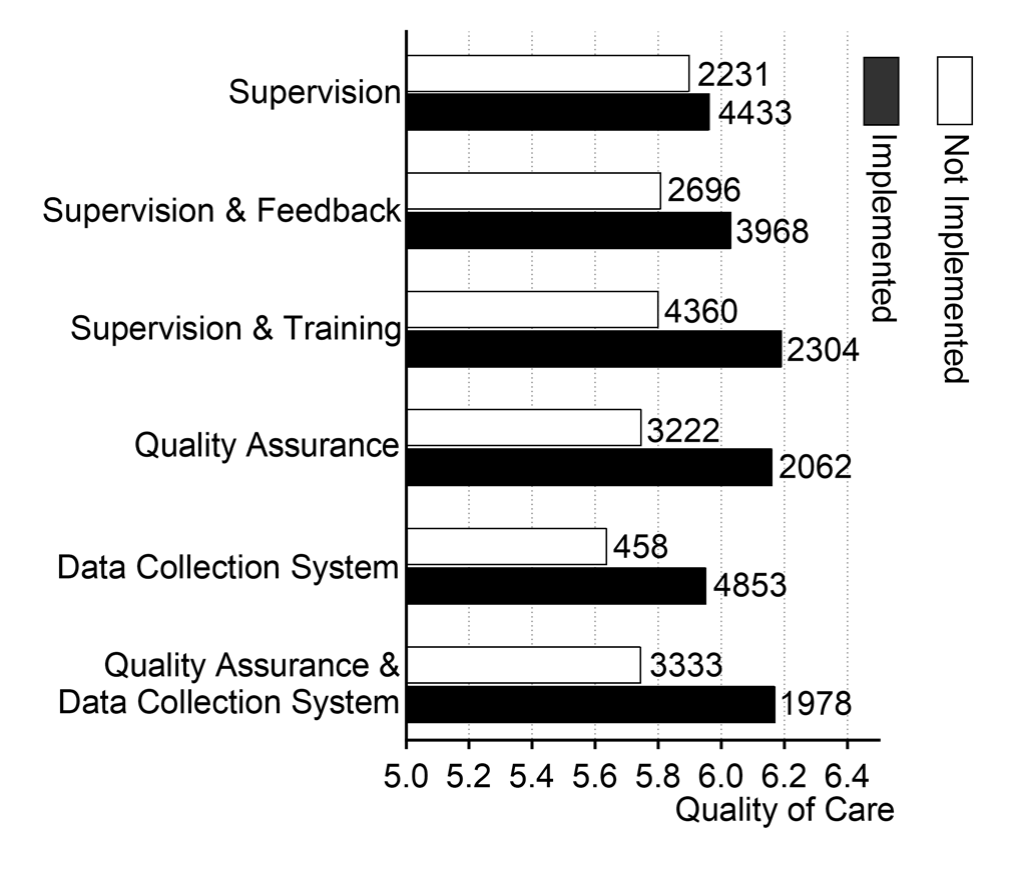
Clinical quality provided by health professionals under various supervision modalities and organisational contexts. The upper three supervision modalities (supervision, supervision plus feedback, and supervision plus training) were directed at health providers, while the lower three organisational contexts (quality assurance, data collection system, and quality assurance plus data collection system) were applied at the facility level. The numbers to the right of each bar indicate how many health providers or facilities implemented the corresponding strategy.

Across all three supervision modalities, regardless of implementation frequency, the group with implemented supervision consistently demonstrated higher quality of care than the group without it. Similarly, health facilities that implemented the context variables reported higher quality of care compared to those that did not (**Figure 1**).

The strategy of supervision alone for health professionals was not significantly associated with quality of care (coefficient = 0.101; 95% confidence interval (CI) = −0.00, 0.20). When supervision was combined with feedback, this strategy was positively associated with quality of care (coefficient = 0.171; 95% CI = 0.07, 0.27). Additionally, supervision, combined with training, showed a significant positive association with quality of care (coefficient = 0.514; 95% CI  = 0.41, 0.62). In terms of the covariates, the child’s age, non-mother caretakers, and non-physicians were negatively associated with the quality of care delivered by health professionals. Conversely, a perceived supportive environment, private ownership, and the health facility’s rural location were positive predictors of higher-quality care (**Table 2**).

**Table 2:** Multilevel regression results of different supervision modalities on clinical quality among health professionals, n = 19 171*

Variables	Supervision only, coefficient (95% CI)	*P*-value	Supervision and feedback, coefficient (95% CI)	*P*-value	Supervision and training, coefficient (95% CI)	*P*-value
Supervision only	0.101 (–0.00, 0.20)	0.55				
Supervision and feedback			0.171 (0.07, 0,27)	<0.001		
Supervision and training					0.514 (0.41, 0.62)	<0.001
Sex						
*Male*	ref		ref		ref	
*Female*	0.027 (–0.03, 0.09)	0.384	0.026 (–0.03, 0.09)	0.391	0.027 (–0.03, 0.09)	0.377
Age of the sick child in months	−0.005 (–0.01, –0.00)	<0.001	−0.005 (–0.01, –0.00)	<0.001	−0.005 (–0.01, –0.00)	<0.001
Relationship to child						
*Mother*	ref		ref		ref	
*Non-mother*	−0.143 (–0.23, –0.06)	<0.01	−0.139 (–0.23, –0.05)	<0.01	−0.144 (–0.23, –0.06)	<0.01
Age of the caretaker	−0.004 (–0.01, 0.00)	0.066	−0.004 (–0.01, 0.00)	0.065	−0.004 (–0.01, 0.00)	0.055
Provider type						
*Physician*	ref		ref		ref	
*Nurse*	−0.363 (–0.52, –0,21)	<0.001	−0.372 (–0.53, –0.21)	<0.001	−0.396 (–0.55, –0.24)	0.001
Years of working	−0.002 (–0.01, 0.01)	0.570	−0.003 (–0.01, 0.00)	0.492	−0.007 (–0.02, 0.01)	0.058
Working hours per day	0.005 (–0.02, 0.03)	0.648	0.005 (–0.02, 0.03)	0.663	0.006 (–0.01, 0.03)	0.584
Years of education	0.012 (–0.01, 0.03)	0.278	0.013 (–0.01, 0.03)	0.246	0.013 (–0.01, 0.03)	0.250
Supportive environment	0.375 (0.27, 0.48)	<0.001	0.365 (0.26, 0.47)	<0.001	0.331 (0.23, 0.44)	<0.001
Ownership						
*Government*	ref		ref		ref	
*Private*	0.258 (0.12, 0.40)	<0.001	0.262 (0.12, 0.40)	<0.001	0.294 (0.15, 0.44)	<0.001
Location						
*Urban*	ref		ref		ref	
*Rural*	0.428 (0.28, 0.58)	<0.001	0.422 (0.27, 0.57)	<0.001	0.408 (0.26, 0.55)	<0.001
Facility type						
*Hospital*	ref		ref		ref	
*Clinic*	−0.064 (–0.22, 0.09)	0.423	−0.072 (–0.23, 0.09)	0.372	−0.101 (–0.26, 0.06)	0.205

CI – confidence interval, ref – reference

*All models adjusted for country dummy variables.

In health facilities lacking quality assurance activities, data collection systems, or both, supervision alone was not significantly related to quality of care. However, in health facilities implementing quality assurance activities, supervision alone was positively associated with quality of care (coefficient = 0.290; 95% CI = 0.14, 0.44). Likewise, supervision alone in facilities that implemented data collection systems was significantly and positively related to quality of care (coefficient = 0.130; 95% CI = 0.02, 0.24). Furthermore, when both quality assurance and data collection systems were present, supervision alone had an even larger positive effect on quality of care compared to facilities that implemented only one strategy (coefficient = 0.319; 95% CI = 0.17, 0.47) (**Table 3**).

**Table 3:** Multilevel regression results on supervision alone of health professionals and clinical quality across different organisational contexts*

Variables	Not implemented quality assurance (n = 10 315), coefficient (95% CI)	*P*-value	Implemented quality assurance (n = 8764), coefficient (95% CI)	*P*-value	Not implemented a data collection system (n = 1346), coefficient (95% CI)	*P*-value	Implemented data collection system (n = 17 825), coefficient (95% CI)	*P*-value	Both not implemented (n = 10 717), coefficient (95% CI)	*P*-value	Both implemented (n = 8454), coefficient (95% CI)	*P*-value
Supervision alone	−0.097 (–0.24, 0.05)	0.250	0.290 (0.14, 0.44)	<0.001	−0.166 (–0.55, 0.21)	0.392	0.130 (0.02, 0.24)	<0.05	−0.111 (–0.25, 0.03)	0.127	0.319 (0.17, 0.47)	<0.001
Sex		<0.001										
*Male*	ref		ref		ref		ref		ref		ref	
*Female*	0.069 (–0.01, 0.15)		−0.028 (–0.12, 0.06)	0.545	0.001 (–0.22, 0.22)	0.992	0.028 –0.03, 0.09)	0.380	0.077 (–0.00, 0.15)	0.055	−0.033 (–0.12, 0.06)	0.483
Age of the sick child in months	−0.005 (–0.01, –0.00)	<0.001	−0.005 (–0.01, –0.00)	<0.01	−0.013 (–0,02, –0.01)	<0.001	−0.004 (–0.01, –0.00)	<0.001	−0.005 (–0.01, –0.00)	<0.001	−0.005 (–0.01, –0.00)	<0.01
Relationship to child												
*Mother*	ref		ref		ref		ref		ref		ref	
*Non-mother*	−0.131 (–0.25, –0.01)	<0.001	−0.157 (–0.29, –0.02)	<0.05	−0.098 (–0.40, 0,20)	0.524	−0.149 (–0.24, –0.06)	<0.01	−0.126 (–0.24, –0.01)	<0.05	−0.154 (–0.29, –0.02)	<0.05
Age of the caretaker	−0.001 (–0.01, 0.00)		−0.007 (–0.01, –0.00)	<0.05	0.001 (–0.01, 0.02)	0.930	−0.004 (–0.01, –0.00)	<0.05	−0.001 (–0.01, 0.00)	0.664	−0.008 (–0.01, –0.00)	<0.05
Provider type												
*Physician*	ref	0.205	ref		ref		ref		ref		ref	
*Nurse*	−0.387 (–0.59, –0.18)	0.250	−0.246 (–0.49, 0.00)	0.051	−0.380 (–1.00, 0.24)	0.228	−0.362 (–0.52, –0.20)	<0.001	−0.366 (–0.57, –0.16)	<0.001	−0.268 (–0.52, –0.02)	<0.05
Years of working	−0.001 (–0.01, 0.01)	<0.001	−0.004 (–0.02, 0.01)	0.530	−0.006 (–0.04, 0.02)	0.696	−0.002 (–0.01, 0.01)	0.598	0.000 (–0.01, 0.01)	0.967	−0.004 (–0.02, 0.01)	0.489
Working hours per day	−0.013 (–0.04, 0.02)		0.030 (–0.00, 0.06)	0.059	0.017 (–0.05, 0.08)	0.595	0.005 (–0.02, 0.03)	0.661	−0.008 (–0.04, 0.02)	0.570	0.028 (–0.00, 0.06)	0.091
Years of education	0.026 (–0.00, 0.06)		−0.003 (–0.04, 0.03)	0.846	0.059 (–0.03, 0.15)	0.197	0.01 (–0.01, 0.03)	0.405	0.026 (–0.00, 0.06)	0.082	−0.003 (–0.04, 0.03)	0.847
Supportive environment	0.502 (0.35, 0.65)	<0.001	0.251 (0.10, 0.40)	<0.01	0.620 (0.19, 1.05)	<0.01	0.367 (0.26, 0.48)	<0.001	0.529 (0.38, 0.67)	<0.001	0.213 (0.06, 0.37)	<0.01
Ownership												
*Government*	ref		ref		ref		ref		ref		ref	
*Private*	0.217 (0.03, 0.41)	<0.001	0.308 (0.08, 0.54)	<0.01	−0.387 (–0.93, 0.16)	0.166	0.316 (0.17, 0.47)	<0.001	0.210 (0.03, 0.39)	<0.05	0.299 (0.06, 0.54)	<0.05
Location												
*Urban*	ref		ref		ref		ref		ref		ref	
*Rural*	0.389 (0.19, 0.59)	0.205	0.556 (0.33, 0.79)	<0.001	0.355 (–0.15, 0.86)	0.169	0.434 (0.28, 0.59)	<0.001	0.390 (0.20, 0.58)	<0.001	0.550 (0.32, 0.78)	<0.001
Facility type		0.250										
*Hospital*	ref	<0.001	ref		ref		ref		ref		ref	
*Clinic*	−0.061 (–0.30, 0.18)		0.081 (–0.15, 0.31)	0.493	0.321 (–0.36, 1.01)	0.357	−0.081 (–0.25, 0.08)	0.332	−0.028 (–0.26, 0.21)	0.816	0.045 (–0.19, 0.28)	0.710

CI – confidence interval, ref – reference

*All models adjusted for country dummy variables.

**Figure 2:**
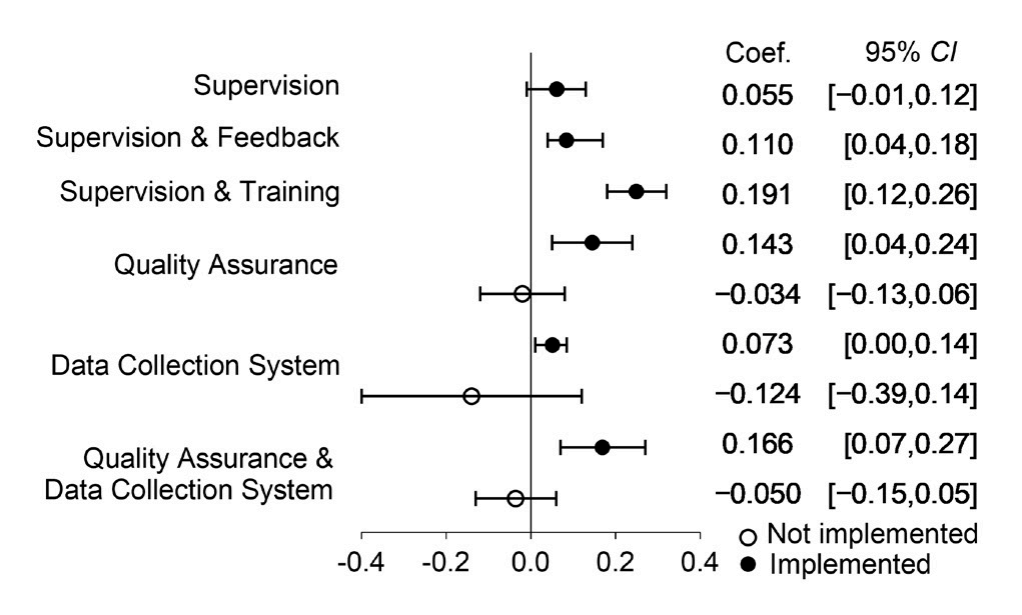
Results of sensitivity analyses on the relationship between various supervision modalities and clinical quality, and between supervision and clinical quality across organisational contexts.

When altering the quality-of-care measurements provided by health professionals, the sensitivity results remained consistent with the previous findings (**Figure 2**; Appendix S2 and S3 in the **Online Supplementary Document**).

## DISCUSSION

In this study, we explored the association between supervision alone and clinical quality, the association between optimised supervision modalities and clinical quality, and the association between supervision alone and clinical quality across different organisational contexts. Our findings indicated that supervision alone was not associated with clinical quality. However, when supervision was optimised through feedback or training, it was significantly related to higher clinical quality. In terms of institutional factors, health facilities that implement data collection systems or quality assurance activities showed a positive relationship between supervision and clinical quality, compared to facilities that did not implement these systems. Furthermore, in facilities that implemented both contexts, the effect of supervision on clinical quality was greater than in facilities that implemented either context alone.

We confirmed that supervision alone, without additional supportive measures, was insufficient to be associated with higher-quality care. Similar research had shown that supervision alone for health professionals was not associated with maternal clinical quality [19]. However, in the same study, supervision alone was found to have a small but significant effect on the quality of care for sick children [19]. This disparity could stem from measuring clinical quality as an average at the health provider level, which might fail to capture the variations in the services provided to different sick children. Moreover, this study did not adjust for the child characteristics, such as age, as covariates, which prior research had shown to be significantly associated with quality of care [34]. Based on these factors, it was rational to conclude that supervision alone, particularly in resource-constrained settings, may not foster the necessary behavioural change or skill improvement required to meet clinical standards.

In contrast to supervision alone, our findings demonstrated that optimised supervision modalities, pairing supervision with training or feedback, were positively associated with improvements in quality of care. Training was one of the most effective ways to enhance the knowledge and technical skills of health professionals, providing them with the tools necessary to deliver higher-quality care [35]. Adequate training ensured that health professionals were equipped to understand and implement best practices, thereby contributing to measurable improvements in patient outcomes and quality of care. Similarly, feedback was the critical component of making supervision effective [36,37]. Constructive feedback could directly facilitate the change process by clarifying performance criteria, identifying gaps in achieving the desired goals, and fostering a supportive relationship between the supervisor and supervisee, thereby encouraging continuous learning and adaptation [38]. This approach was consistent with the Deming cycle of continuous quality improvement (plan-do-check-act), which highlighted the importance of iterative learning and feedback in driving lasting improvements [39].

Institutional contexts are pivotal in enhancing the effectiveness of supervision. In facilities equipped with data collection systems or quality assurance activities, supervision alone can be positively correlated with the quality of care. Data collection systems provide supervisors with critical performance data, improving the efficiency and quality of supervision [5,20]. Similarly, quality assurance activities have been shown to improve the effectiveness of supervision by ensuring a focus on quality improvement and establishing a framework for continuous monitoring [30]. When facilities implemented data collection systems or quality assurances, they created an environment conducive to effective supervision. Prior research has also indicated that cultivating a workplace culture that supports interventions was key to successful implementation [27,40]. Therefore, to avoid inefficient use of supervision resources, support contexts at the institutional level were necessary to nudge supervision toward greater effectiveness. In this study, over 91% of facilities have data collection systems, while fewer than 40% have quality assurance activities. In LMICs, the first step should be to ensure the widespread implementation of these systems across all health facilities. With a strong data system in place, the next step should focus on improving data utilisation, such as sharing information among health professionals, patients, and institutions. For quality assurance, initial efforts should focus on auditing performance and key indicators, with future activities expanding to total quality management, addressing workforce, patient care, resources, and organisational efficiency. Concrete practices should align with the local health system to account for country-specific challenges and resources.

In resource-limited areas, particularly in LMICs, implementing supervision alone might exacerbate strain on already limited resources. Our findings suggested that supervision became more effective when coupled with optimised modalities like feedback or training. Health facilities should also focus on fostering a quality-oriented culture, ensuring adequate preparedness for implementing supervision, and driving its effectiveness. Facilities that invested in improving the practices of health professionals and in promoting a quality-oriented, well-equipped electronic system were more likely to become learning organisations or high-reliability organisations. These facilities not only benefited from improved supervision outcomes but also developed the capacity to adapt to changes and reduce medical errors [41,42]. Overall, implementing these strategies had the potential to enhance the effectiveness of supervision and contribute to long-term quality improvements.

An interesting finding emerges regarding the covariates – a more supportive environment perceived by health professionals is positively associated with higher-quality care. A supportive environment is characterised by a clear job description, defined promotion opportunities, and performance incentives, all of which are recognised as factors that promote the quality of care [43]. When health professionals have well-defined responsibilities and opportunities for development, they are better able to realise their full potential. Moreover, an organisational culture that emphasises a supportive environment is linked to improved organisational innovation and performance [44]. A supportive environment enhances the intrinsic motivation of health professionals, empowering them to perform at a higher level, while external supervision ensures that this motivation is translated into sustained high performance. The interaction between a supportive environment and supervision fosters the continuous development of health professionals, further enhancing their overall performance.

Despite these important findings, several limitations must be acknowledged. First, our conclusions were drawn from data on sick child healthcare services in LMICs, and caution should be exercised when generalising these results to other healthcare settings or service areas. Second, the clinical quality assessed in this study represents a process quality indicator. While high clinical quality was a critical prerequisite for improved patient health outcomes, future research should explore the direct impact of supervision on patient outcomes to provide a more comprehensive understanding. Additionally, the measurement of clinical quality was based on the simple summation. Future research could explore specific sub-domains of clinical quality or apply weighted indicators in the questionnaire to refine the measurement. Next, supervision and optimised supervision modalities were measured using binary indicators, which fail to capture the extent of supervision implementation. Future research could refine the measurement of supervision by evaluating its frequency, intensity, and quality using questionnaires or interviews with health professionals. Lastly, this study did not assess health professionals’ attitudes toward supervision, which might be a potential factor influencing the effective implementation of supervision. Further research could explore these intangible factors to provide deeper insights into the successful implementation of supervision.

## CONCLUSIONS

In this study, we explored the relationship between supervision alone and clinical quality in child healthcare services, emphasising the role of optimised supervision modalities and supportive organisational contexts in enhancing supervision’s effectiveness. Our findings indicate that supervision alone is not associated with better clinical quality. However, when paired with strategies like feedback or training, supervision becomes significantly more effective. Additionally, health facilities with supportive contexts – such as data collection systems and quality assurance activities – play a crucial role in enhancing the effectiveness of supervision. To maximise the impact of supervision in resource-limited settings, it is important to combine supervision with training or feedback, develop robust electronic health systems, and promote a culture of quality. Future research should focus on understanding the underlying soft factors, such as health professionals’ attitudes and motivation, that may influence the effectiveness of supervision and its role in improving clinical quality.

## Additional material


Online Supplementary Document
